# Prevalence of *Leptospira* serogroups in buffaloes from the Brazilian Amazon

**DOI:** 10.1002/vms3.271

**Published:** 2020-04-21

**Authors:** Israel B. Guedes, Gisele O. de Souza, Lilian A. R. de Oliveira, Juliana F. de P. Castro, Antônio F. de Souza Filho, Anderson L. P. Maia, Marcos B. Heinemann

**Affiliations:** ^1^ Laboratório de Zoonoses Bacterianas Departamento de Medicina Veterinária Preventiva e Saúde Animal Faculdade de Medicina Veterinária e Zootecnia Universidade de São Paulo São Paulo Brazil; ^2^ Médico Veterinário Auditor Fiscal Agropecuário Agência de Defesa e Inspeção Agropecuária do Estado do Amapá Brazil

**Keywords:** Amazonia, Brazil, Buffalo, *Leptospira*, MAT

## Abstract

Although Brazil has one of the largest buffalo populations in the Americas, buffalo leptospirosis is still poorly explored when compared to that in bovines; thus, the aim of this research was to carry out a large serological study for leptospirosis in this species in the Brazilian Amazon. For this, we collected 1,405 serum samples from buffaloes raised in the Amazon delta region, which is considered a major area of buffalo production in Brazil. The test used was a microscopic agglutination test (MAT) adopting 34 *Leptospira* antigens, some of which have never been tested for buffaloes in Brazil, including autochthonous strains; in total, 20 serogroups were evaluated. From the total of 1,405 serum samples, 894 (63.6%) reacted in the MAT to at least one of the 20 serogroups, and 511 (36.4%) did not react. The serogroups Sejroe, Autumnalis and Pomona were the most prevalent, with titres ranging from 100 to 12,800, and the autochthonous strains used were not significant in relation to the reference serovars. Leptospirosis in buffaloes seems to have a serological profile similar to leptospirosis in cattle, mainly due to the prevalence of the Sejroe serogroup; however, the results of this study suggested that in the Brazilian Amazon, *Leptospira* strains that are serologically distinct from the autochthonous strains isolated in the southeastern region of Brazil may be circulating in these animals. Other serovars could also be inserted into the panel of antigens used in MAT for serological studies on buffaloes.

## Graphical abstract

Buffalo production in Brazil is one of the fastest growing activities, not just for food or employment but also as a cultural factor, mainly in Amazonia, which leads to close contact between these animals and humans. The serology performed revealed that in the Brazilian Amazon, leptospirosis was present in buffaloes and seemed to have a unique profile, especially due to the possible presence of leptospires that were serologically distinct from the leptospires isolated from other animals in Brazil. The results found in this study provided information about buffalo leptospirosis in Brazil, which is still poorly explored in the country compared to bovine leptospirosis.

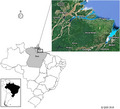

## 1 INTRODUCTION

Buffaloes are considered tolerant animals and can be used for various types of livestock production; they have been prospected as possible alternatives for food production and agriculture in less developed regions of the world (Desta, 2012). This trend has occurred specifically because buffaloes perform better than cattle in reproductive parameters and have the ability to convert low quality food (Bernardes, 2007), even in flooded areas such as in the floodplain of the Amazon River, where buffalo productivity is greater than that from raising bovines (Sheikh, Merry, & McGrath, 2006). Although these advantages are attributed to buffaloes, there are still many gaps in knowledge regarding the genetics, zootechnical characteristics and rusticity of these animals (El Debaky et al., 2019).

By 2,000, the buffalo population in Brazil had become the most crescent herd of the world (Vale, Minervino, Neves, Morini, & Coelho, 2013), and in recent years, it has come to have an effective population of approximately 950,000 head. It should be highlighted that approximately 50% of these animals are concentrated in the northern region of the country, more specifically in the area known as Amazon delta, which comprises part of the Amapá and Pará states, including Marajó Island (IBGE & Instituto Brasileiro de Geografia e Estatística, 2017). The activity has become so important in the region that in Amapá, there more buffalo that are slaughtered than cattle (Soares, Esteves, Fariam, Texeira, & Araujos, 2014).

Leptospirosis appears to manifest in buffaloes in the same way as in cattle, with special emphasis on reproductive disorders such as abortion (Balakrishman, Meenambigai, & Roy, 2014; Marianelli et al., 2007), but there have also been reports of jaundice (Upadhye, Rajasekhar, Ahmed, & Krishnappa, 1983) and mastitis (Ahmed, 1990). The isolation of *Leptospira* in urine from a healthy buffalo (Vasconcellos et al., 2001) as well as the detection through PCR of bacteria in the urine of asymptomatic buffaloes show that these animals can also become reservoirs of leptospires in the environment by elimination through the urine (Denipitiya, Chandrasekharan, Abeyewickreme, Hartskeerl, & Hapugoda, 2017).

The serological studies conducted in Brazil for buffaloes involve various sample sizes and number of serovars are used as antigens in the panel of the microscopic agglutination test (MAT), which is generally the same standard used for most other animal species. A study carried out in São Paulo state that used 24 antigens in the MAT panel revealed a prevalence that 43.7% of buffaloes were reactive in a total of 879 animals examined (Favero et al., 2002); in Vale do Ribeira, São Paulo state, 37.7% of positive results were found in 403 animals assessed for only 10 antigens with the MAT (Langoni, Fava, Cabral, Silva, & Chagas, 1999); in Vale do Ribeira, another study of 222 buffaloes showed a that 50.9% of animals were tested positive with the use of 24 antigens in the MAT (Fujii, Kasai, Vasconcellos, Richtzenhain, & Cortez, 2001). In the Amazon region, a study verified a prevalence of 34.37% in 256 animals examined for 25 antigens with the MAT (Oliveira, Silva, Pinheiro, & Langoni, 2013), and in 212 samples of buffaloes examined in Pará state, 80.0% of the animals were reactive in a MAT with a panel of 27 antigens (Viana et al., 2009).

The main purpose of this study was to add knowledge to buffalo leptospirosis in Brazil through a serological study conducted in the Brazilian Amazon using an expanded panel of antigens in MAT, including some reference serovars and autochthonous strains isolated in Brazil never tested before for buffaloes.

## 2 MATERIALS AND METHODS

**Figure 1:**
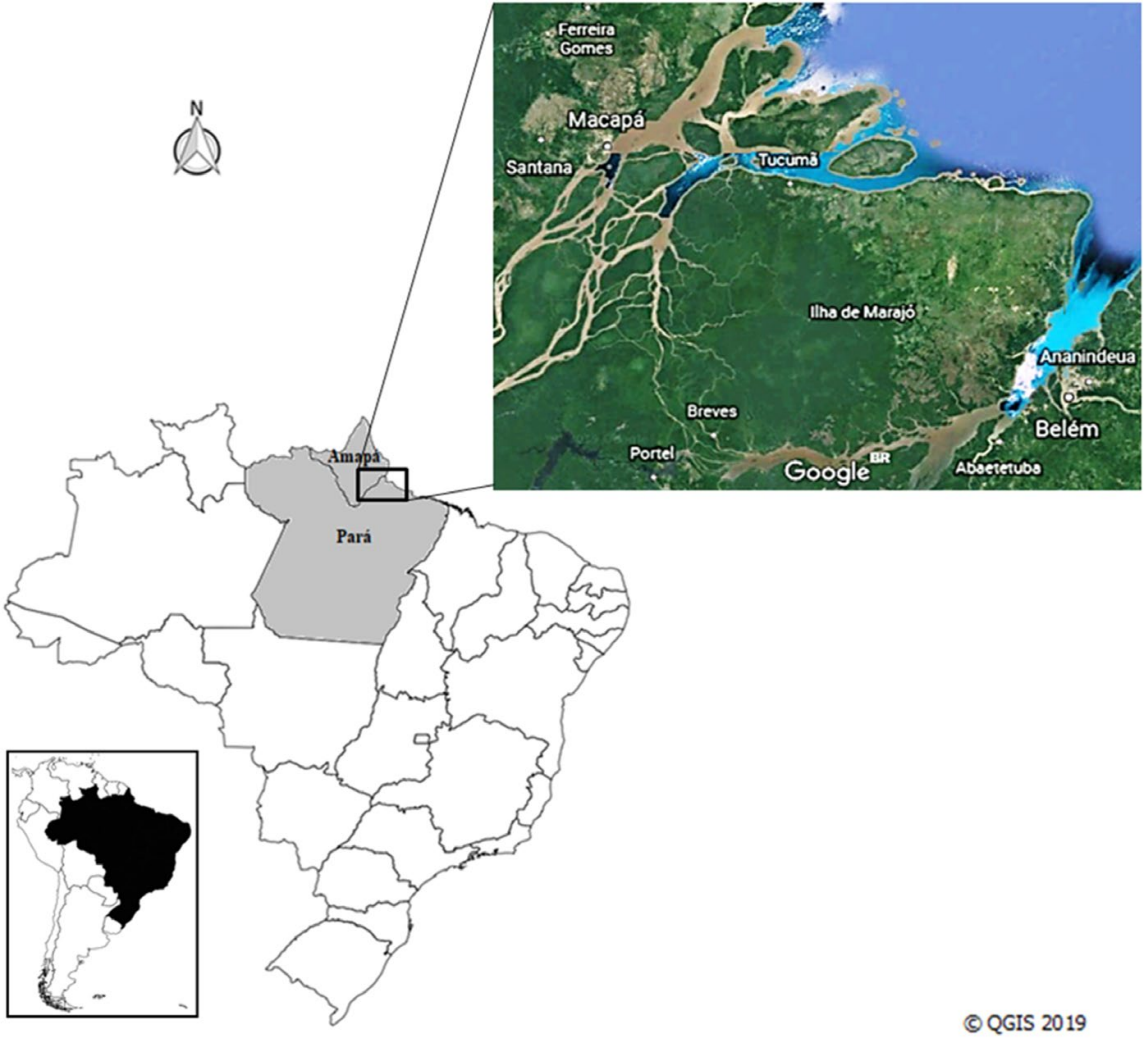
Geographical location of the Amazon delta in Brazil

This work used the region known as the Amazon delta as the study area, where the great Amazon River flows into the Atlantic Ocean in extreme northern Brazil, between the states of Pará and Amapá (Figure 1). This region has a humid equatorial climate characterized by high temperatures (average of 26ºC) and high rainfall throughout the year (2,300 mm) as well as tropical vegetation that shelters a great diversity of plant and animal species (Fisch, Marengo, & Nobre, 1998). One unique aspect of this region is the large flood areas with daily variations in the river water level (Vogt et al., 2016), which favours the buffalo production in extensive systems, especially for meat production (Soares et al., 2014).

In January 2019, we collected blood from 1,405 buffaloes slaughtered at a slaughterhouse located in the Macapá microregion, Amapá state, which receives animals from different farms in the Amazon delta. The sampling was by convenience, and the animals were predominantly from the river buffalo group (the Murrah, Mediterranean and Jafarabadi breeds), composed by males and females of at least 12 months of age that had the ability to produce meat and that lacked records of vaccination against leptospirosis in addition to unknown reproductive situations.

The microscopic agglutination test (MAT) was performed following Faine, Adler, Bolin, and Perolat (1999) and employed a panel of 34 live antigens that represented 20 different serogroups; these antigens were reference serovars and autochthonous strains isolated in Brazil (Table 1).

**Table 1:** Serovars of *Leptospira* spp. used as antigens in the microscopic agglutination test (MAT) listed by serogroups

Serogroup	Serovar
Australis	Australis
	Bratislava
Autumnalis	Autumnalis
	Butembo
Ballum	Castellonis
Bataviae	Brasiliensis^a^, ^b^
Canicola	Canicola
Celledoni	Whitcombi
Cynopteri	Cynopteri
Djasiman	Sentot
Grippotyphosa	Grippotyphosa
	Bananal^a^, ^c^
Hebdomadis	Hebdomadis
Icterohaemorrhagiae	Copenhageni
	Icterohaemorrhagiae
Javanica	Javanica
Mini	Mini
Panama	Panama
Pomona	Pomona
	Pomona (GR6) ^a^, ^d^
Pyrogenes	Pyrogenes
Ranarum	Ranarum^a^, ^e^
Sejroe	Gorgas
	Guaricura^a^, ^f^
	Hardjo‐prajitno
	Hardjo‐bovis
	Medanensis
	Polonica
	Recreo
	Ricardi
	Sejroe
	Wolffi
Shermani	Shermani
Tarassovi	Tarassovi

^a^ Autochthonous strains isolated in Brazil.

^b^ Santa Rosa, Sulzer, and Pestana de Castro (1972).

^c^ Marvulo et al. (2002).

^d^ Miraglia et al. (2008).

^e^ Yasuda et al. (1986).

^f^ Vasconcellos et al. (2001).

Statistical analysis was carried out through descriptive statistics using frequency measures. For general prevalence, all reactive samples were considered at a 1:100 dilution, and for determination of the most prevalent serogroups, the ranking technique was used, which considered the only reactive samples for the antigen that had the highest titre; animals that were reactive to more than one antigen with a predominant titre were disregarded for this analysis (Vasconcellos et al., 1997).

## 3 RESULTS

Of all 1,405 serum samples, 894 (63.6%) reacted in the MAT to at least one of the 20 serogroups, and 511 (36.4%) did not react. Considering only the reactive samples by ranking technique (665/894), the titres ranged from 100 to 12,800 (Table 2); the serogroups Sejroe, Autumnalis and Pomona were most prevalent, whereas no reactions were detected for the serogroups Bataviae, Canicola, Celledoni, Javanica, Pyrogenes, Mini and Shermani.

**Table 2:** Frequency of antibody titres found for 13 serogroups of *Leptospira* spp. in relation to the number of reactive samples by the ranking technique

Serogroups	Titres									
	100	200	400	800	1,600	3,200	6,400	12,800	Total	Total (%)
Sejroe	49	99	96	26	4	—	—	—	274	41.2
Autumnalis	43	71	36	3	—	—	—	—	153	23.0
Pomona	15	40	25	20	4	4	—	1	109	16.3
Grippotyphosa	7	15	11	4	3	—	—	—	40	6.0
Tarassovi	6	12	4	4	1	—	1	—	28	4.2
Ranarum	10	14	3	—	—	—	—	—	27	4.1
Sentot	11	1	—	—	—	—	—	—	12	1.8
Hebdomadis	5	3	—	—	—	—	—	—	8	1.2
Panama	1	3	—	—	—	—	—	—	4	0.6
Ballum	—	1	1	1	—	—	—	—	3	0.5
Icterohaemorrhagiae	—	2	1	—	—	—	—	—	3	0.5
Cynopteri	2	—	1	—	—	—	—	—	3	0.5
Australis	—	1	—	—	—	—	—	—	1	0.1

**Figure 2:**
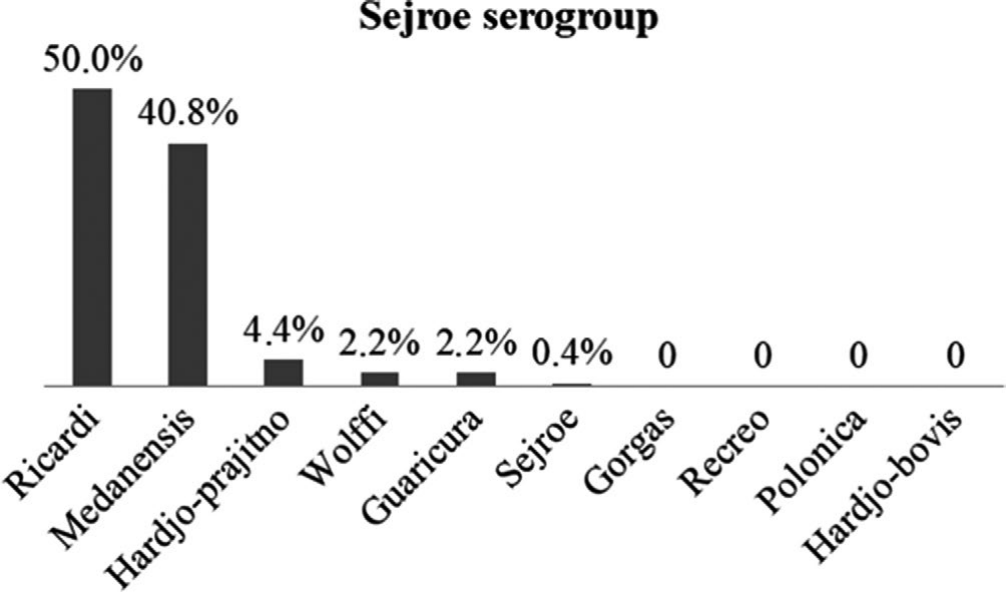
Percentage of reactive samples for each serovar representative of the Sejroe serogroup used in relation to number of reactive for this serogroup

The Sejroe serogroup had the highest number of serovars tested, revealing a very different reaction profile among the serovars. Ricardi and Medanensis were the most prevalent inside the Sejroe serogroup (Figure 2). The serovars Hardjo‐prajitno, Wolffi and Guaricura, which are widely used as representatives of this serogroup, had reaction percentage lower than 5.0%.

**Figure 3:**
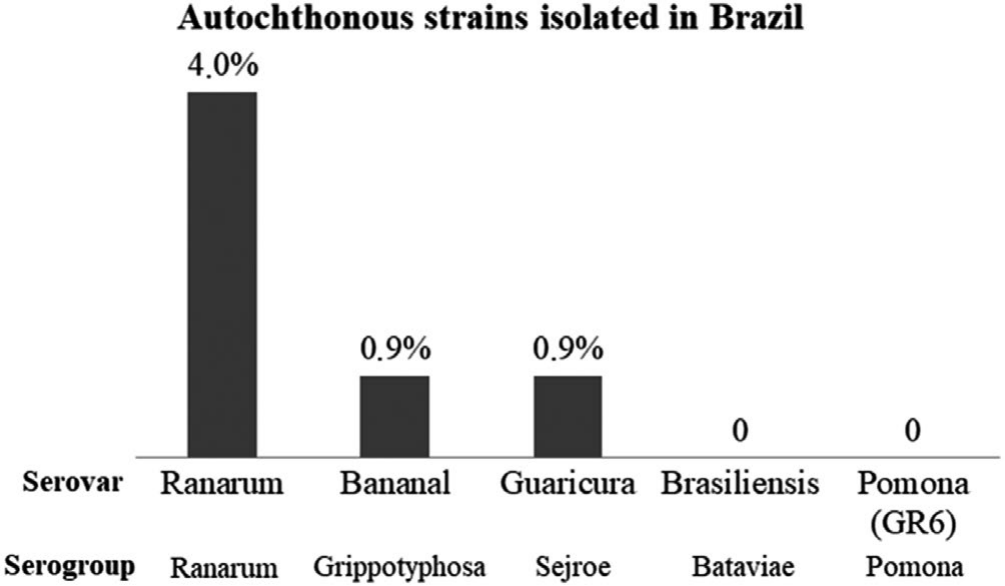
Percentage of reactive samples for each autochthonous strain used in relation to total number of reactive samples by the ranking technique

Among the autochthonous strains isolated in Brazil that were used in this study, serovar Ranarum had the highest number of reactions, while the others were not expressive (Figure 3). All autochthonous serovars used were isolated from animals in the southeastern region of Brazil as there have thus far been no isolates from the Brazilian Amazon.

## 4 DISCUSSION

Leptospirosis in buffaloes has been reported in several countries through serological studies that indicate different prevalences, such as 14.6% (33/226) in buffalo raised in Trinidad, Caribbean (Adesiyun, Hull‐Jackson, Clarke, Whittington, & Seepersadsingh, 2009); 22.2% (111/500) in Argentina (Konrad et al., 2013); 25% (3/12) in Egypt (Felt et al., 2011); 48% (81/170) in the Philippines (Villanueva et al., 2016); 58.73% (111/189) in Iran (Hajikolaei, Ghorbanpour, & Abdollapour, 2006); and 88.8% (111/125) in India (Selvaraj et al., 2010). In Thailand, a high prevalence of leptospirosis in buffaloes has been reported (Tangkanakul, Smits, Jatanasen, & Ashford, 2005; Wongpanit, Suwanacharoen, & Srikram, 2012), which has become a public health concern in some regions of Asia, such as Sri Lanka, where a large part of the economy is based on agriculture that relies on rice cultivation in flooded fields and uses buffaloes in their activities, implying a possible relationship of these animals with human leptospirosis in the country (Agampodi et al., 2011). The use of an expanded antigen panel in the MAT may have increased the sensitivity of the test for this work, but the prevalence found in this study (63.6%) showed that leptospirosis was present in the buffaloes of the Brazilian Amazon. This still deserves attention from a public health perspective, since in the Amazonia, it is common to use these animals for food through the consumption of meat and milk. In addition, buffaloes are part of the local culture and are also used in both labour and tourism, causing closer contact with humans (Barreto, Lobato, Pereira, & Serra, 2017). A limitation that occurred in this study was the logistical conditions at the slaughterhouse, because during the slaughter of the animals it was not possible to realize an association between the characteristics of each animal and epidemiological information with it is respective serum sample.

Water is one of the major source of leptospirosis contamination (Faine et al., 1999), and this could reflect one of the risk factors for buffaloes, since in addition to their gregarious habits, they tend to seek out water for wallowing and bathing, mainly in hot weather, with the objectives of thermoregulation and protection from ecto‐parasites (Napolitano, Pacelli, Grasso, Braghieri, & Rosa, 2013). In the Amazon, it is also common to observe buffaloes in rivers and dammed areas along with wild animals, mainly those of semi‐aquatic habits, such as capybaras (*Hydrochaeris hydrochaeris*), which have already been reported to able to eliminate leptospires through urine (Marvulo et al., 2009).

The Sejroe serogroup was the most prevalent in the animals, which indicated a similarity with bovine leptospirosis in Brazil, since this serogroup was the most prevalent (Sarmento et al., 2012; Pinna et al., 2018), especially serovar Hardjo, which has been isolated in Brazil from cases of leptospirosis in cattle (Chiareli et al., 2012; Chideroli et al., 2016). In buffaloes, the serovar Hardjo also stands out within the Sejroe serogroup (Favero et al., 2002; Rocha, Lima, Paz, Langoni, & Moraes, 2019; Viana et al., 2009), disagreeing with the results of the present study, where the serovars Ricardi and Medanensis were the antigens responsible for the prevalence of the Sejroe serogroup over the others, since 33.5% of the samples were reactive only for the serovars Ricardi and/ or Medanensis, against 5.0% of the samples reactive only for the serovars Hardjo‐prajitno, Hardjo‐bovis, Guaricura and/ or Wolffi (data not shown).

The first report of serovar Ricardi isolation occurred in a patient in Malaysia (Alexander, Evans, Toussaint, Marchwicki, & McCrumb, 1957), and it was subsequently isolated from intraocular samples of horses with recurrent uveitis (Hartskeerl et al., 2004), which suggested that this serovar could be circulating in animals. Serovar Medanensis was first described in Indonesia, where it was isolated from a healthy dog (Kouwenarr & Wolff, 1929) and has rarely been reported in the literature; consequently, serological reactions occurring for medanensis are considered cross‐reactions with serovar Hardjo (Loewenstein, McLachlan‐Troup, Hartley, & English, 2008). These serovars belong to two distinct subgroups within the Sejroe serogroup. Serovar Ricardi is inserted in subgroup Saxkoebing, and serovar Medanensis was introduced into subgroup Wolffi along with serovars Hardjo, Gorgas, Wolffi, Recreo and Guaricura, among others. There exists a third subgroup, called Sejroe, which includes the serovars Sejroe and Polonica (Kmety, 1977). Neither serovars has ever been used in serological studies in Brazil to represent the Sejroe serogroup.

In Brazil, it was agreed to use the strains of serovars Hardjo and serovar Wolffi in the MAT panel to represent the Sejroe serogroup; however, if only these serovars were used in this work, the Sejroe serogroup would not have been the most prevalent because few reactions were detected for the cited antigens. In the same way, serovar Guaricura, strain M4/98, which is isolated from the urine of a healthy buffalo in São Paulo (Vasconcellos et al., 2001), was also included in panel of antigens used in the MAT after having been observed as one of the most prevalent serovars in cattle in the central‐western and southeastern regions of Brazil (Sarmento et al., 2012); however, this antigen no longer seemed to be representative for animals in other areas of the country, such as buffaloes in northeastern Brazil (Oliveira et al., 2018) and in cattle in the Amazon (Guedes et al., 2019). This evidence reinforced the hypothesis of regional variability in the strains, influenced by climatic and environmental issues (Plank & Dean, 2000), especially in a country as extensive and heterogeneous as Brazil. The inclusion of serovars Ricardi and Medanensis into the panel of antigens used in the MAT to perform serological studies on buffaloes should be evaluated, particularly with the aim of increasing reactions to the Sejroe serogroup. In addition, although cross‐reactions among serovars of the same serogroup happen in serology tests (Faine et al., 1999), the possibility that these two serovars are circulating in the buffaloes of the Amazon region cannot be excluded, therefore, further studies should also be encouraged to evaluate this condition, mainly aimed at the isolation of leptospires from these animals.

The Autumnalis serogroup was the second most prevalent; in a similar manner to the trend seen in the Sejroe serogroup, this trend seemed to be influenced by the antigens that represented it in MAT. In this study, serovar Butembo was involved in 98.7% of reactions within the Autumnalis serogroup (data not shown). In other countries, the Autumnalis serogroup was not expressive at serology for buffalo leptospirosis, as antigens different other than those of the Butembo serovar were used to represent this serogroup (Adesiyun et al., 2009; Balakrishnan, Padian, Roy, & Chandran, 2015; Konrad et al., 2013). In Brazil, serovar Butembo appears to be related to rodents and wild animals (Lilenbaum, 1996) and is widely used in serology; in cattle, it has been reported as one of the most prevalent serovars (Tonin et al., 2010) and is also associated with reproductive disorders in animals (Saldanha et al., 2007).

The Pomona serogroup followed the most prevalent serogroups, as described in buffaloes in the northeastern region of Brazil, making this serogroup one of the most dominant (Pimenta et al., 2019). In the present work, the autochthonous strain GR6 (Pomona), which was isolated from a slaughtered pig in São Paulo (Miraglia et al., 2008), did not generate reactions detected in MAT for serogroup Pomona, and all reactions for these serogroups were derived from the reference Pomona antigen, in contrast with what has already been reported in cattle in Amazonia, where all reactions detected for the Pomona serogroup were obtained by the GR6 strain (Guedes et al., 2019).

Among all five of the autochthonous strains isolated from animals in Brazil that were used in this study, the Ranarum sample obtained the largest number of reactions. This strain was isolated from the kidney of an aborted equine fetus and was characterized serologically as belonging to the saprophytic species of *Leptospira*, but it was not possible to conclude whether this was the cause of the abortion (Yasuda, Sulzer, Giorgi, & Soares, 1986). The use of this antigen had the aim of evaluating the behaviour of the Ranarum serogroup in buffalo leptospirosis in Brazil, since it shares saprophytic and pathogenic strains and had not yet been tested in the country for this animal species; in Thailand, serovar Ranarum was part of the panel of antigens used in MAT and was found to be one of the most prevalent in buffaloes (Chadsuthi et al., 2017). In cattle in Malaysia, it surpassed even the Sejroe serogroup (Suwancharoen, Chaisakdanugull, Thanapongtharm, & Yoshida, 2013).

The introduction of locally isolated strains into the MAT panel has been reported as a way to increase the sensitivity of this technique to detect leptospirosis‐positive animals (Sarmento et al., 2012); in some cases, this method results in more reactions for locally isolated strains than for the reference strains, that end up being standardized on the MAT from region to region (Daud et al., 2018). Diverging from this trend, the present study found that autochthonous strains isolated in southeastern Brazil were not representative of the serology regarding the increase in reactions in general prevalence, as was observed in a serological study for cattle carried out in the Brazilian Pantanal, where nine strains isolated from different regions of Brazil were used and reacted at very low percentages (Miashiro et al., 2018).

In conclusion, this study showed that the Sejroe serogroup was the most prevalent in buffaloes, as in cattle; however, serovars Ricardi and Medanensis were the most prevalent in this serogroup, surpassing the Hardjo and Wolffi antigens traditionally used in Brazil, and could be included in the MAT panel to evaluate the true participation of these antigens as representatives of the Sejroe serogroup. In addition, the autochthonous strains isolated in the southeastern region of Brazil that were used in this study were not relevant, promoting the assumption that other local and serologically distinct strains may be circulating in buffaloes of the Amazon region.

## CONFLICT OF INTEREST

The authors declare that they have no conflict of interest.

## AUTHOR CONTRIBUTION

Israel Barbosa Guedes: Conceptualization; Investigation; Methodology; Writing‐review & editing. Gisele Oliveira de Souza: Methodology. Lilian Abgail Ribeiro de Oliveira: Methodology. Juliana Fernandes de Paula Castro: Methodology. Antonio Souza Filho: Methodology. Anderson Luiz Pinheiro Maia: Methodology. Marcos Bryan Heinemann: Conceptualization; Investigation; Project administration; Validation; Writing‐review & editing. 

## ETHICAL APPROVAL

This work was approved by the Ethics Committee on Animal Use of the School of Veterinary Medicine and Animal Science (Universidade de São Paulo) – CEUA/FMVZ nº 5,613,211,118.

## References

[vms3271-bib-0001] Adesiyun, A. A. , Hull‐Jackson, C. , Clarke, N. , Whittington, C. , & Seepersadsingh, N. (2009). Leptospirosis in water buffalo (*Bubalus bubalis*) in Trinidad. Veterinarski Arhiv, 79, 77–86.

[vms3271-bib-0002] Ahmed, R. (1990). Leptospiral infection in lactating buffaloes. Pakistan Veterinary Journal, 10, 98–99.

[vms3271-bib-0003] Alexander, A. D. , Evans, L. B. , Toussaint, A. J. , Marchwicki, R. H. , & McCrumb, F. R. Jr (1957). Leptospirosis in Malaya Il. Antigenic Analysis of 110 leptospiral strains and other serologic studies. The American Journal of Tropical Medicine and Hygiene, 6, 871–889. 13470208

[vms3271-bib-0004] Balakrishman, G. , Padian, S. S. , Roy, P. , & Chandran, D. J. (2015). Seroprevalence of leptospirosis among buffaloes from Tamil Nadu. Indian Journal of Veterinary Research, 24, 15–16.

[vms3271-bib-0005] Balakrishnan, G. , Meenambigai, T. V. , & Roy, P. (2014). Diagnosis of Bovine Leptospirosis by 16s rRNA Based Polymerase Chain Reaction. Indian Journal of Field Veterinarians, 10, 87–88.

[vms3271-bib-0006] Barreto, E. O. , Lobato, A. S. , Pereira, P. V. V. , & Serra, D. R. O. (2017). Caracterização do Turismo de Base Comunitária em Polos Turísticos do Estado do Pará. Revista Brasileira De Ecoturismo, 10, 113–127. 10.34024/rbecotur.2017.v10.6620

[vms3271-bib-0007] Bernardes, O. (2007). Buffaloes breeding in Brasil. Italian Journal of Animal Science, 6, 162–167. 10.4081/ijas.2007.s2.162

[vms3271-bib-0008] Chadsuthi, S. , Bicout, D. J. , Wiratsudakul, A. , Suwancharoen, D. , Petkanchanapong, W. , Modchang, C. , … Chalvet‐Monfray, K. (2017). Investigation on predominant *Leptospira* serovars and its distribution in humans and livestock in Thailand, 2010–2015. PLoS Neglected Tropical Diseases, 11, e0005228 10.1371/journal.pntd.0005228 28182662 PMC5325611

[vms3271-bib-0009] Chiareli, D. , Cosate, M. R. V. , Moreira, E. C. , Leite, R. C. , Lobato, F. C. F. , Silva, J. A. , … Marcelino, A. P. (2012). Controle da leptospirose em bovinos de leite com vacina autógena em Santo Antônio do Monte, Minas Gerais. Pesquisa Veterinária Brasileira, 32, 633–639. 10.1590/S0100-736X2012000700008

[vms3271-bib-0010] Chideroli, R. T. , Pereira, U. P. , Gonçalves, D. D. , Nakamura, A. Y. , Alfieri, A. A. , Alfieri, A. F. , & Freitas, J. C. (2016). Isolation and molecular characterization of *Leptospira borgpetersenii* serovar Hardjo strain Hardjobovis in the urine of naturally infected cattle in Brazil. Genetics and Molecular Research, 15, 7 10.4238/gmr.15018473 26909976

[vms3271-bib-0011] Daud, A. , Fuzi, N. M. H. M. , Arshad, M. M. , Kamarudin, S. , Mohammad, W. M. Z. W. , Amran, F. , & Ismail, N. (2018). Leptospirosis seropositivity and its serovars among cattle in Northeastern Malaysia. Veterinary World, 11, 840–844. 10.14202/vetworld.2018.840-844 30034179 PMC6048083

[vms3271-bib-0012] Denipitiya, D. T. H. , Chandrasekharan, N. V. , Abeyewickreme, W. , Hartskeerl, R. A. , & Hapugoda, M. D. (2017). Identification of cattle, buffaloes and rodents as reservoir animals of *Leptospira* in the District of Gampaha, Sri Lanka. Biomed Central Research Notes, 10, 5 10.1186/s13104-017-2457-4 28330498 PMC5363019

[vms3271-bib-0013] Desta, T. T. (2012). Introduction of domestic buffalo (*Bubalus bubalis*) into Ethiopia would be feasible. Renewable Agriculture and Food Systems, 27, 305–313. 10.1017/S1742170511000366

[vms3271-bib-0014] El Debaky, H. A. , Kutchy, N. A. , Ul‐Husna, A. , Indriastuti, R. , Akhter, S. , Purwantara, B. , & Memili, E. (2019). Review: Potential of water buffalo in world agriculture: Challenges and opportunities. Applied Animal Science, 35, 255–268. 10.15232/aas.2018-01810

[vms3271-bib-0015] Faine, S. , Adler, B. , Bolin, C. , & Perolat, P. S. (1999). Leptospira and leptospirosis (2nd ed). Melbourne: Medi. Sci.

[vms3271-bib-0016] Favero, A. C. M. , Pineiro, S. R. , Vasconcellos, S. A. , Morais, Z. M. , Ferreira, F. , & Ferreira Neto, J. S. (2002). Sorovares de leptospiras predominantes em exames sorológicos de bubalinos, ovinos, caprinos, eqüinos, suínos e cães de diversos Estados brasileiros. Ciência Rural, 32, 613–619. 10.1590/S0103-84782002000400011

[vms3271-bib-0017] Fisch, G. , Marengo, J. A. , & Nobre, C. A. (1998). Uma revisão geral sobre o clima da Amazônia. Acta Amazônica, 28, 101–126. 10.1590/1809-43921998282126

[vms3271-bib-0018] Fujii, T. U. , Kasai, N. , Vasconcellos, S. A. , Richtzenhain, L. J. , & Cortez, A. (2001). Anticorpos anti‐*Neospora caninum* e contra outros Agentes de Abortamentos em Búfalas da Região do Vale do Ribeira, São Paulo, Brasil. Arquivos do Instituto Biológico, 68, 5–9.

[vms3271-bib-0019] Guedes, I. B. , Araújo, S. A. D. A. , de Souza, G. O. , de Souza Silva, S. O. , Taniwaki, S. A. , Cortez, A. , … Heinemann, M. B. (2019). Circulating *Leptospira* species identified in cattle of the Brazilian Amazon. Acta Tropica, 191, 212–216. 10.1016/j.actatropica.2019.01.011 30639452

[vms3271-bib-0020] Hajikolaei, M. R. H. , Ghorbanpour, M. , & Abdollapour, G. (2006). Seroprevalence of Leptospiral Infection in Buffalo (*Bubalus bubalis*). Bulletin‐ Veterinary Institute in Pulawy, 50, 341–344.

[vms3271-bib-0021] Hartskeerl, R. A. , Goris, M. G. A. , Brem, S. , Meyer, P. , Kopp, H. , Gerhards, H. , & Wollanke, B. (2004). Classification of *Leptospira* from the eyes of horses suffering from recurrent uveitis. Journal of Veterinary Medicine Series B, 51, 110–115. 10.1111/j.1439-0450.2004.00740.x 15107036

[vms3271-bib-0022] IBGE, Instituto Brasileiro de Geografia e Estatística . (2017). Censo Agro 2017, Resultados preliminares, Bubalinos/Brasil. Retrieved from https://censoagro2017.ibge.gov.br/templates/censo_agro/resultadosagro/pecuaria.html?localidade=0&tema=75659

[vms3271-bib-0023] Kmety, E. (1977). Studium antigennej structury leptospir. Klasifikacia serologickej skupiny Hebdomadis. Folia Facultatea Medicina Universitate Comenianae Bratislava, 2, 245–309.

[vms3271-bib-0024] Konrad, J. L. , Campero, L. M. , Caspe, G. S. , Brihuega, B. , Draghi, G. , Moore, D. P. , … Campero, C. M. (2013). Detection of antibodies against *Brucella abortus*, *Leptospira* spp., and Apicomplexa protozoa in water buffaloes in the Northeast of Argentina. Tropical Animal Health and Production, 45, 1751–1756. 10.1007/s11250-013-0427-y 23765549

[vms3271-bib-0025] Kouwenaar, W. , & Wolff, J. W. (1929). Honden als leptospirendragers. Nederlandsch Indische Bladen Voor Diergeneeskunde, 41, 457–465.

[vms3271-bib-0026] Langoni, H. , Del Fava, C. , Cabral, K. G. , Silva, A. V. , & Chagas, S. A. P. (1999). Aglutininas Antileptospíricas em Búfalos do Vale do Ribeira, Estado de São Paulo. Ciência Rural, 29, 305–307. 10.1590/S0103-84781999000200019

[vms3271-bib-0027] Lilenbaum, W. (1996). Atualização em leptospirose bovina. Revista Brasileira De Medicina Veterinária, 18, 9–13.

[vms3271-bib-0028] Loewenstein, L. , McLachlan‐Troup, T. , Hartley, M. , & English, A. (2008). Serological survey for evidence of *Leptospira interrogans* in free‐living platypuses (*Ornithorhynchus anatinus*). Australian Veterinary Journal, 86, 242–245. 10.1111/j.1751-0813.2008.00305.x 18498563

[vms3271-bib-0029] Marianelli, C. , Tarantino, M. , Astarita, S. , Martucciello, A. , Capuano, F. , & Galiero, G. (2007). Molecular detection of *Leptospira* species in aborted fetuses of water buffalo. Veterinary Record, 161, 310–311. 10.1136/vr.161.9.310 17766812

[vms3271-bib-0030] Marvulo, M. F. V. , Paula, C. D. , Ferreira, P. M. , Morais, Z. M. , Delbem, A. C. B. , Fávero, A. C. M. , … Verdade, L. M. (2002). Detection of *Leptospira* in two free living populations of capybaras (*Hydrochaeris hydrochaeris*) from São Paulo State, Brazil. International Leptospirosis Society, Bridgetown, 62.

[vms3271-bib-0031] Marvulo, M. F. V. , Silva, J. C. R. , Ferreira, P. M. , Morais, Z. M. , Moreno, A. M. , Doto, D. S. , … Neto, J. S. F. (2009). Experimental leptospirosis in capybaras (*Hydrochaeris hydrochaeris*) infected with *Leptospira interrogans* serovar Pomona. Journal of Zoo and Wildlife Medicine, 40, 726–730. 10.1638/2007-0042.1 20063819

[vms3271-bib-0032] Miashiro, A. F. , Vasconcellos, S. A. , Morais, Z. M. D. , Souza, G. O. D. , Leal Filho, J. M. , Figueiredo, A. D. O. , & Pellegrin, A. O. (2018). Prevalência de leptospirose em rebanhos bovinos no Pantanal de Mato Grosso do Sul. Pesquisa Veterinária Brasileira, 38, 41–47. 10.1590/1678-5150-pvb-4992

[vms3271-bib-0033] Miraglia, F. , Moreno, A. M. , Gomes, C. R. , Paixão, R. , Liuson, E. , Morais, Z. M. , … Vasconcellos, S. A. (2008). Isolation and characterization of *Lepospira interrogans* form pigs slaughtered in São Paulo State, Brazil. Brazilian Journal of Microbiology, 39, 501–507. 10.1590/S1517-83822008000300017 24031254 PMC3768438

[vms3271-bib-0034] Napolitano, F. , Pacelli, C. , Grasso, F. , Braghieri, A. , & De Rosa, G. (2013). The behaviour and welfare of buffaloes (*Bubalus bubalis*) in modern dairy enterprises. Animal, 7, 1704–1713. 10.1017/S1751731113001109 23803231

[vms3271-bib-0035] Oliveira, G. C. , Silva, D. B. , Pinheiro, V. L. C. , & Langoni, H. (2013). Pesquisa de Anticorpos anti‐Leptospíricos em bubalino. Ars Veterinária, 29, 33 10.15361/2175-0106.2013v29n4p33

[vms3271-bib-0036] Oliveira, P. R. F. D. , Soares, L. B. F. , Borges, J. D. M. , Barrosa, N. D. C. , Langoni, H. , Brandespim, D. F. , … Mota, R. A. (2018). Occurrence of serological reactions for serogroup Sejroe (CTG and Prajtino) in female buffalo in the state of Pernambuco, Brazil. Brazilian Journal of Microbiology, 49, 795–800. 10.1016/j.bjm.2018.02.007 29609849 PMC6175753

[vms3271-bib-0037] Pimenta, C. L. R. M. , Bezerra, C. D. S. , Morais, D. D. A. , Silva, M. L. C. R. , Nogueira, D. B. , Costa, D. F. D. , … Azevedo, S. S. D. (2019). Seroprevalence and predominant serogroups of *Leptospira* sp. in serological tests of ruminants in northeastern Brazil. Semina: Ciências Agrárias, 40, 1512–1522. 10.5433/1679-0359.2019v40n4p1513

[vms3271-bib-0038] Pinna, M. H. , Martins, G. M. , Loureiro, A. P. , & Lilenbaum, W. (2018). Detection of bovine carriers of *Leptospira* by serological, bacteriological, and molecular tools. Tropical Animal Health and Production, 50, 883–888. 10.1007/s11250-018-1512-z 29349716

[vms3271-bib-0039] Plank, R. , & Dean, D. (2000). Oveview of the epidemiology, microbiology, and pathogenesis of Leptospira spp. in humans. Microbes and Infection, 2, 1265–1276. 10.1016/S1286-4579(00)01280-6 11008116

[vms3271-bib-0040] Rocha, K. S. , Lima, M. S. , Paz, G. S. , Langoni, H. , & Moraes, C. C. G. (2019). Detecção de anticorpo anti‐*Brucella* sp. e anti‐ *Leptospira* spp. em búfalos (*Bubalus bubalis*) abatidos em matadouro na cidade de. Belém, Pará. Revista De Ciências Agrárias, 62(4), 10.22491/rca.2019.3046.

[vms3271-bib-0041] Saldanha, G. B. , Cavazini, N. C. , Silva, A. S. , Fernandes, M. B. , Badke, M. R. T. , & Pivetta, C. G. (2007). Sorologia positiva para *Leptospira butembo* em bovinos apresentando problemas reprodutivos. Ciência Rural, 37, 1182–1184. 10.1590/S0103-84782007000400046

[vms3271-bib-0042] Santa Rosa, C. A. , Sulzer, C. R. , & Pestana de Castro, A. F. (1972). A new leptospiral serotype in the Bataviae group, isolated in Sao Paulo, Brasil. American Journal of Veterinary Research, 33, 1719–1721. 5047124

[vms3271-bib-0043] Sarmento, A. M. C. , Azevedo, S. S. , Morais, Z. M. , Souza, G. O. , Oliveira, F. C. S. , Gonçalves, A. P. , … Vasconcellos, S. A. (2012). Emprego de estirpes *Leptospira* spp. isoladas no Brasil na microtécnica de soroaglutinação microscópica aplicada ao diagnóstico da leptospirose em rebanhos bovinos de oito estados brasileiros. Pesquisa Veterinária Brasileira, 32, 601–606. 10.1590/S0100-736X2012000700003

[vms3271-bib-0044] Selvaraj, J. , Murali Manohar, B. , Govindarajan, R. , Jayakumar, V. , Meenambigai, T. V. , & Balachandran, C. (2010). Seroprevalence of leptospirosis in she‐buffaloes (*Bos bubalis*) at slaughter in Chennai, India. Buffalo Bulletin, 29, 95–97.

[vms3271-bib-0045] Sheikh, P. A. , Merry, F. D. , & McGrath, D. G. (2006). Water buffalo and cattle ranching in the Lower Amazon Basin: Comparisons and conflicts. Agricultural Systems, 87, 313–330. 10.1016/j.agsy.2005.02.003

[vms3271-bib-0046] Soares, B. V. , Esteves, C. , Fariam, P. B. , Texeira, J. T. , & Araujos, T. S. (2014). Perfil de bovídeos abatidos nos matadouros oficiais do Estado do Amapá, Brasil. Publicações Em Medicina Veterinária E Zootecnia, 8, 12 10.22256/pubvet.v8n12.1731

[vms3271-bib-0047] Suwancharoen, D. , Chaisakdanugull, Y. , Thanapongtharm, W. , & Yoshida, S. (2013). Serological survey of leptospirosis in livestock in Thailand. Epidemiology and Infection, 141, 2269–2277. 10.1017/S0950268812002981 23308397 PMC9151404

[vms3271-bib-0048] Tangkanakul, W. , Smits, H. L. , Jatanasen, S. , & Ashford, D. A. (2005). Leptospirosis: An emerging health problem in Thailand. Southeast Asian Journal of Tropical Medicine and Public Health, 36, 281–288. 15916031

[vms3271-bib-0049] Thevanesam, V. , Burns, M. A. , Agampodi, S. B. , Palihawadana, P. , Thaipadungpanit, J. , Perera, S. , … Peacock, S. J. (2011). Leptospirosis outbreak in Sri Lanka in 2008: Lessons for assessing the global burden of disease. The American Society of Tropical Medicine and Hygiene, 85, 471–478. 10.4269/ajtmh.2011.11-0276 PMC3163869 21896807

[vms3271-bib-0050] Tonin, A. A. , Azevedo, M. I. , Escobar, T. P. , Casassola, I. , Santos, L. G. , Silva, A. S. , … Badke, M. R. T. (2010). Leptospirose Bovina: Aumento na incidência da *Leptospira interrogans* sorovar butembo no rebanho do Estado de Santa Catarina, Brasil. Acta Veterinaria Brasilica, 4, 294–297.

[vms3271-bib-0051] Upadhye, A. S. , Rajasekhar, M. , Ahmed, S. N. , & Krishnappa, G. (1983). Isolation of *Leptospira andaman* from an active case of jaundice in a Buffalo. Indian Veterinary Journal, 60, 319–320.

[vms3271-bib-0052] Vale, W. G. , Minervino, A. H. H. , Neves, K. A. L. , Morini, A. C. , & Coelho, J. A. S. (2013). Buffalo under Threat in Amazon Valley, Brazil. Buffalo Bulletin, 32, 121–131.

[vms3271-bib-0053] Vasconcellos, S. A. , Barbarini Junior, O. , Umehara, O. , Morais, Z. M. , Cortez, A. , Pinheiro, S. R. , … Ferreira Neto, J. S. (1997). Leptospirose bovina. Níveis de ocorrência e sorotipos predominantes em rebanhos dos Estados de Minas Gerais, São Paulo, Rio de Janeiro, Paraná, Rio Grande do Sul e Mato Grosso do Sul, no período de janeiro a abril de 1996. Arquivos do Instituto Biológico, 64, 7–15.

[vms3271-bib-0054] Vasconcellos, S. A. , Oliveira, J. C. F. , Morais, Z. M. , Baruselli, P. S. , Amaral, R. , Pinheiro, S. R. , … Hartskeerl, R. A. (2001). Isolation of *Leptospira santarosai*, serovar Guaricura from buffaloes (*Bubalus bubalis)* in Vale do Ribeira, São Paulo, Brazil. Brazilian Journal of Microbiology, 32, 298–300. 10.1590/S1517-83822001000400008

[vms3271-bib-0055] Viana, R. B. , Del Fava, C. , Moura, A. C. B. , Cardoso, E. C. , De Araújo, C. V. , Monteiro, B. M. , … Vasconcellos, S. A. (2009). Ocorrência de Anticorpos anti‐ *Neospora caninum*, *Brucella* sp. e *Leptospira* spp. em Búfalos (*Bubalus bubalis*) criados na Amazônia. Arquivos do Instituto Biológico, 76, 453–457. 10.13140/2.1.1966.1446

[vms3271-bib-0056] Villanueva, M. A. , Mingala, C. N. , Gloriani, N. G. , Yanagihara, Y. , Isodas, N. , Nakajima, C. , … Koizumi, N. (2016). Serological investigation of *Leptospira* infection and its circulation in one intensive‐type water buffalo farm in the Philippines. Japanese Journal of Veterinary Research, 64, 15–24. 27348885

[vms3271-bib-0057] Vogt, N. , Pinedo‐Vasquez, M. , Brondízio, E. S. , Rabelo, F. G. , Fernandes, K. , Almeida, O. , … Dou, Y. (2016). Local ecological knowledge and incremental adaptation to changing flood patterns in the Amazon delta. Sustainability Science, 11, 611–623. 10.1007/s11625-015-0352-2

[vms3271-bib-0058] Wasfy, M. O. , El‐Bassiouny, A. A. , Murray, C. K. , Boshra, M. , Hatem, M. E. , El‐Tras, W. F. , … Pimentel, G. (2011). Cross‐Species Surveillance of *Leptospira* in Domestic and Peri‐Domestic Animals in Mahalla City, Gharbeya Governorate, Egypt. The American Society of Tropical Medicine and Hygiene, 84, 420–425. 10.4269/ajtmh.2011.10-0393 PMC3042818 21363980

[vms3271-bib-0059] Wongpanit, K. , Suwanacharoen, D. , & Srikram, A. (2012). Serological Survey of Leptospirosis in Thai Swamp Buffalo (*Bubalus bubalis*) in Sakon Nakhon Province, Thailand. Kasetsart Journal – Natural Science, 46, 736–741.

[vms3271-bib-0060] Yasuda, P. H. , Sulzer, C. R. , Giorgi, W. , & Soares, M. E. G. (1986). *Leptospira biflexa* sorotipo *ranarum* isolado de feto abortado de equino. Revista De Microbiologia, 17, 25–27.

